# Combating the challenges of COVID-19 pandemic: Insights into molecular mechanisms, immune responses and therapeutics against SARS-CoV-2

**DOI:** 10.1093/oxfimm/iqad001

**Published:** 2023-01-10

**Authors:** Kriti Negi, Meetu Agarwal, Isha Pahuja, Bhavya Bhardwaj, Mansi Rawat, Ashima Bhaskar, Ved Prakash Dwivedi

**Affiliations:** Immunobiology Group, International Centre for Genetic Engineering and Biotechnology, New Delhi, India; Department of Molecular Medicine, Jamia Hamdard University, New Delhi, India; Immunobiology Group, International Centre for Genetic Engineering and Biotechnology, New Delhi, India; Department of Molecular Medicine, Jamia Hamdard University, New Delhi, India; Immunobiology Group, International Centre for Genetic Engineering and Biotechnology, New Delhi, India; Immunobiology Group, International Centre for Genetic Engineering and Biotechnology, New Delhi, India; Immunobiology Group, International Centre for Genetic Engineering and Biotechnology, New Delhi, India; Immunobiology Group, International Centre for Genetic Engineering and Biotechnology, New Delhi, India

**Keywords:** SARS-CoV-2, COVID-19, vaccines, immunotherapy, diagnosis

## Abstract

Severe acute respiratory syndrome coronavirus 2 (SARS-CoV-2) infection causes lethal coronavirus disease (COVID-19). SARS-CoV-2 has been the chief source of threat to public health and safety from 2019 to the present. SARS-CoV-2 caused a sudden and significant rise in hospitalization due to respiratory issues and pneumonia. We are consistently uncovering new information about SARS-CoV-2, and yet so much is to explore to implement efficient interventions to combat the emergent variants and spread of the ongoing pandemic. Information regarding the existing COVID-19 pandemic is streamlining continuously. However, clinical symptoms of SARS-CoV-2 infections spanning from asymptomatic infection to severe death-instigating disease remain consistent with preliminary reports. In this review, we have briefly introduced highlights of the COVID-19 pandemic and features of SARS-CoV-2. We have focused on current knowledge of innate and adaptive immune responses during SARS-CoV-2 infections and persisting clinical features of recovered patients. Furthermore, we have discussed how these immune responses are not tightly regulated and imbalance can direct the latter phases of COVID-19, long-COVID symptoms, and cause detrimental immunopathogenesis. COVID-19 vaccines are also discussed in detail to describe the efforts going around the world to control and prevent the infection. Overall, we have summarized the current knowledge on the immunology of SARS-CoV-2 infection and the utilization of that knowledge in the development of a suitable COVID-19 therapeutics and vaccines.

## Introduction

In the past two decades, coronaviruses (CoVs) have been linked to consequential disease outbursts in East Asia and Middle Eastern countries. Severe acute respiratory syndrome (SARS) and Middle East respiratory syndrome (MERS) started to make an appearance in 2002 and 2012 respectively. Lately, a novel CoV (nCoV) that is SARS-COV-2 emerged in December 2019 in Wuhan, China and caused mayhem all across the world, touching every continent while becoming a global threat to the human population [1]. CoVs are a member of coronaviridae, types of which infect a broad range of hosts, and the symptoms could cover anything from the common cold to severe and at last fatal illnesses. SARS-CoV-2 is one of the seven members of the CoV cluster that infects humans. CoVs are comparatively larger in size with a diameter of 60–140 nm and characteristic spikes in the range of 9–12 nm. These spikes are responsible for the solar corona appearance of the virions [2]. At first, the evidence of early investigations on the interspecies viral transmission from animals to humans depicted that the coronavirus disease (COVID-19) virus could have been cross transmitted from bats to humans, the genomic analysis of SARS-COV-2 showed 96% similarity to the virus extracted from bats (Beta/CoV/RaTG13/2013) [3]. The evidence of predominant panicle of COVID-19 patients had initial major symptoms such as cough, fever, cold, dyspnea, etc. with a specific population having an underlined medical condition [1]. Various clinical research studies have shown that more bilateral patchy shadows and ground-glass opacity were observed in the lungs in consideration of higher mortality rate [4]. Asymptomatic expression of SARS-COV-2 possesses a greater risk of transmission since screening measures like temperature checks would not be helpful to corroborate the virus. By now, it was very clear that the virus can be transmitted easily and could possess asymptomatically, all the countries were put under quarantine with restricted to no travelling norms, in consideration of its speed of transmission, increasing mortality rate, and to our dismay, new viral strains are emerging with a variety of new symptoms causing strenuous situation among the population. However, by the utilization of the most advanced technologies around the world, researchers have made it possible to get a hold of this virus by discovering new ways and technology to test and prevent the virus at the earliest, and now with so many promising COVID-19 vaccine candidates, hopes of getting our normal lives back are very high. However, the emergence of new variants globally still persists as a threat worldwide. Evidence from long-term clinical studies will further corroborate correlates of immune protection from emerging variant strains of SARS-CoV-2 [5]. Further strategies to counteract persisting as well as emerging variants must be devised to end global COVID-19 pandemic.

## Emergencies and transmission of SARS-COV-2

SARS-CoV-2 has spread worldwide emanating from Wuhan, China and continues to thrive. This all started when several incidences of pneumonia of unknown etiology were encountered in Wuhan, China [1]. Clinical symptoms in hospitalized patients exhibited resemblance to SARS and MERS infections [2]. Moreover, unresponsiveness to antibiotic treatment was indicative of virus-induced pneumonia. Clinical samples from seven patients with severe pneumonia were analyzed to determine the cause of the disease, out of which five samples were positive for CoVs. Furthermore, metagenomic analysis using next-generation sequencing (NGS) of patient sample (WIV04) isolated from bronchoalveolar lavage (BAL) fluid confirmed 2019-nCoV (presently called SARS-CoV-2) as the cause of the ongoing COVID-19 outbreak [6].

Apart from the involvement of intermediate host animals in transmitting COVID-19 disease to humans, not much was known initially regarding modes of SARS-CoV-2 transmission. Until cases unrelated to the seafood market and transmission beyond China was reported, transmission among individuals was deemed improbable. Insight into the transmission dynamics of COVID-19 is continuously advancing. To the best of our current knowledge, various factors have been linked with the global spread of SARS-CoV-2 infection, the highly contagious nature of COVID-19 being a major driver of transmission. In this section, we have summarized potential modes of transmission for SARS-CoV-2 responsible for the spread of the disease.

**Figure 1.**
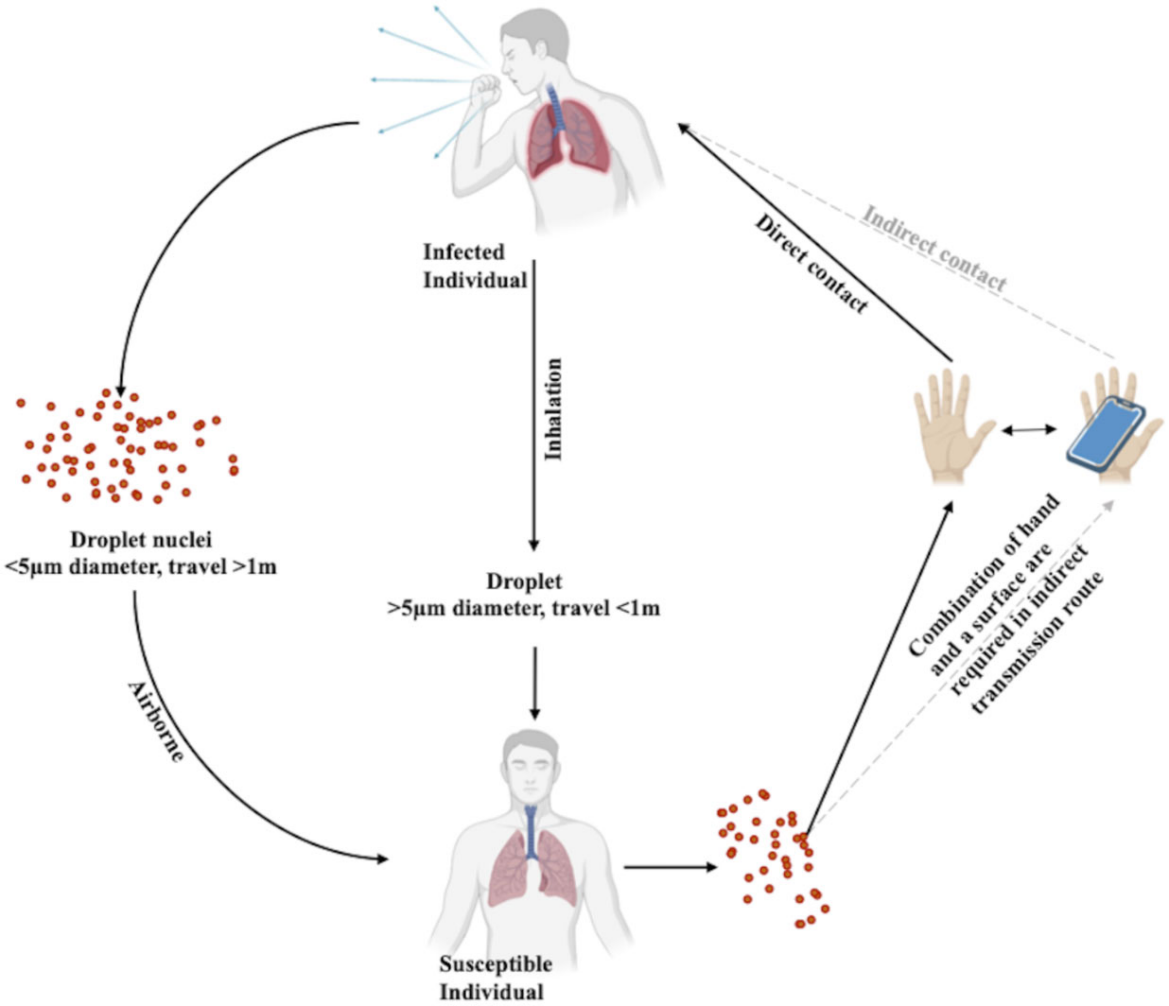
Transmission of SARS-CoV-2 through dispersal of virus-laden discharge or through direct unprotected contact with infected patients.

COVID-19 is transmittable among individuals through direct unprotected contact with infected patients or through expelled contaminated droplets (Fig. 1) (Report of the WHO-China Joint Mission on Coronavirus Disease 2019). Infected individuals via physical actions like sneezing, coughing or speaking can transmit SARS-CoV-2 to individuals in the vicinity (within 1 m) (Fig. 1) [7]. On investigating 75 465 COVID-19 cases in China, it was established that transmission of SARS-CoV-2 occurs majorly via close contact [Report of the WHO-China Joint Mission on Coronavirus Disease 2019 (COVID-19)]. Analogous to SARS-CoV and MERS-CoV, SARS-CoV-2 primarily impacts the respiratory tract and is widely propagated through respiratory secretions. The spread of SARS-CoV-2 occurs by infected individuals through dispersal of virus-laden droplets (>5 to 10 µm) and aerosols (≤5 µm) that persist as an infectious intermediary when suspended in air over long distances and time (Fig. 1) (transmission of SARS-CoV-2: implications for infection prevention precautions). Airborne dispersion of expiratory ejecta of diseased individuals upon inhalation can lead to the spread of COVID-19 among close contacts. Numerous physical actions can result in the formation of virus-laden aerosols by infected patients, which upon airborne dispersal can come in contact with susceptible individuals and result in infection. In clinical settings, aerosol-forming procedures like nebulization, bronchoscopy, cardiopulmonary resuscitation, etc. are major sources of airborne transmission of SARS-CoV-2-loaded aerosols (CDC 2020). Distinct studies have demonstrated that aerosolized SARS-CoV-2 retained infectivity in the aerosols for up to 3–16 h [8, 9], thus aerosols can accumulate over time, contaminate proximate milieu and transmit COVID-19. Furthermore, SARS-CoV-2 can be transmitted through extra-pulmonary routes by infected individuals. Distinct studies have reported the manifestation of SARS-CoV-2 RNA in feces and urine samples of COVID-19 patients [10, 11]. Manifestation of nCoV in biological samples like urine and feces of patients has been ascertained by anal swabs and viral cultures [12]. Examination of patient stool samples yielded persistent viral shedding in contrast to nasopharynx and oropharynx samples, even after the resolution of the clinical symptoms and radiological findings [13]. Additionally, SARS-CoV-2 transmission via urine was validated in an asymptomatic patient with urine sample positive for viral nucleic acid [14]. Besides refining our knowledge about COVID-19 spread, this information necessitates the need to include diverse biological samples for diagnosis. The presence of SARS-CoV-2 RNA has also been reported in patient’s plasma, serum samples [12]. However, due to the presence of relatively low viral titers, possibility of bloodborne transmission is highly unlikely. So far, there is no indication of transmission of SARS-CoV-2 from infected pregnant women to offspring via intrauterine or transplacental route. Further assessment is required to ascertain if this remains valid. According to WHO, it is definite that viable virus cannot be transmitted by breastfeeding to an infant (breastfeeding and COVID-19). Another mode of transmission of SARS-CoV-2 is indirect or fomite transmission. Biological discharge by an infected person like respiratory emissions can give rise to fomites or infected surfaces and lead to environmental contamination [8, 15]. Physical contact with fomites can lead to viral transmission in the proximate milieu and the consequent spread of COVID-19. Depending on the environmental conditions (temperature, humidity), SARS-CoV-2 can remain viable on diverse surfaces for periods ranging from hours to days [8]. Therefore, COVID-19 can spread indirectly by coming in contact with surfaces contaminated with the nCoV.

We are consistently uncovering new information about SARS-CoV-2 and still there is so much to explore about transmission dynamics to implement efficient interventions to combat the spread of the ongoing pandemic.

## Virology and pathogenesis of SARS-COV-2

### SARS-CoV-2 versus SARS-CoV-1: infection and load dynamics

The SARS-CoV-2 virology is accountable for the fatal infectivity of the COVID-19 disease. The enhanced infectivity of SARS-CoV-2 can be justified by the structural changes on the surface proteins of SARS-CoV-2, which is responsible for stronger binding to angiotensin-converting enzyme 2 (ACE2) receptors. SARS-CoV-2 infectiousness in the respiratory tract is highest within the first 5 days when symptoms are observed as the viral load is highest during this period. On the contrary, CoV-1 load is prominent during the second week of infection. This reason is responsible for nominal contagiousness of the disease in the first week. Also, this assists in the early detection of SARS-CoV-2 infection, which was utilized in the pandemic to develop better diagnostics [16].

### Receptors and infection

**Figure 2.**
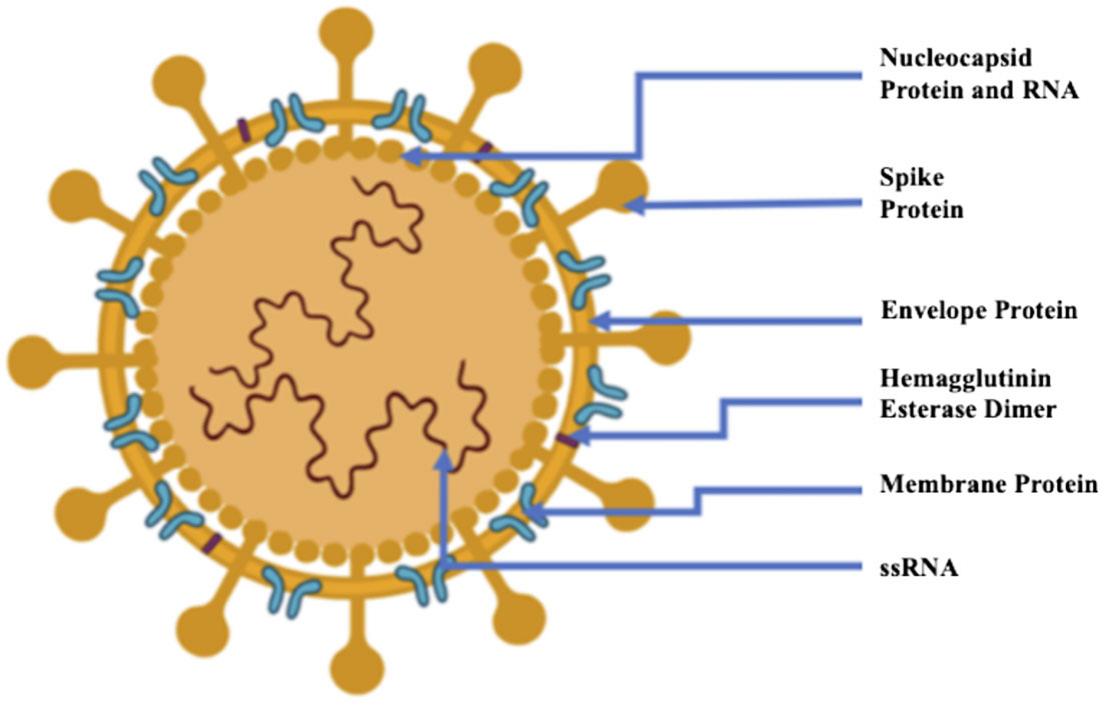
Diagrammatic representation of structural organization of CoVs.

Viruses interact with the host cell receptor and attach to the cell surface. This is followed by penetration into the cells where these viruses replicate and assemble themselves. The released virus particles attack other healthy cells. The structural proteins that are constituents of CoVs are: nucleocapsid (N), envelope (E), membrane (M) and spike (S) (Fig. 2). All of these proteins are required to produce structurally complete viral particles. However, recently, it has been found that CoVs can be infective even if it is not assembled completely, suggesting that these CoVs might encode proteins with compensatory functions or either containing dispensable proteins [17–19]. Independently, each protein is involved in structure formation of virus particles along with other aspects of the replication cycle. Such as, S protein is responsible for attachment to host cell receptors followed by fusion and then entry into the host cell (pre-fusion structure of a human CoV spike protein—PubMed). This S protein is also known to mediate cell–cell fusion between infected and uninfected cells forming syncytia or multinucleated cells, which allows direct spreading of viral bodies by overthrowing the neutralizing antibodies (nABs) [20, 21]. The protein N is involved in processes related to the making of nucleocapsids, viral genome along with replicative cycle and host cellular response to viral bodies [22, 23]. Remarkably, the localization of N protein in the endoplasmic reticulum (ER)–Golgi (GI) region probably reveals their role in assembly and budding [24, 25]. However, transient expression of N protein causes a substantial increase in virus-like particle (VLP) production, suggesting their requirement for complete virion formation instead of limited to envelope formation [26, 27]. The viral envelope shape is defined by the most abundant structural M protein [28]. This M protein is also known as a central organizer for CoV assembly, which interacts with all other major structural proteins of CoV [29]. The major force behind virion envelope formation is homotypic interactions between the M proteins, which alone is not sufficient for the formation of virion. The interaction of S and M proteins is necessary for S protein retention in the ER–GI complex and its incorporation in new virions [30], whereas the interaction between M and N proteins is responsible for stabilization of nucleocapsid as well as the virion’s internal core leading to viral assembly completion [31, 32]. The M and E protein interaction causes the production and release of VLPs [33–35]. Among all these proteins, the E protein is known to be the smallest structural protein with enigmatic properties. As E protein is exclusively expressed during the replication cycle in the infected cells and a small portion is merged into the virion envelope [36]. Most of these proteins are localized at intracellular trafficking sites, viz. ER, GI, ER–GI complex (ERGIC), where they play a role in CoV assembly formation and budding [37]. The E protein is essential in the production and maturation of virus as its absence has led to reduced viral titres, incompetent progeny and crippled viral maturation [38, 39].

**Figure 3.**
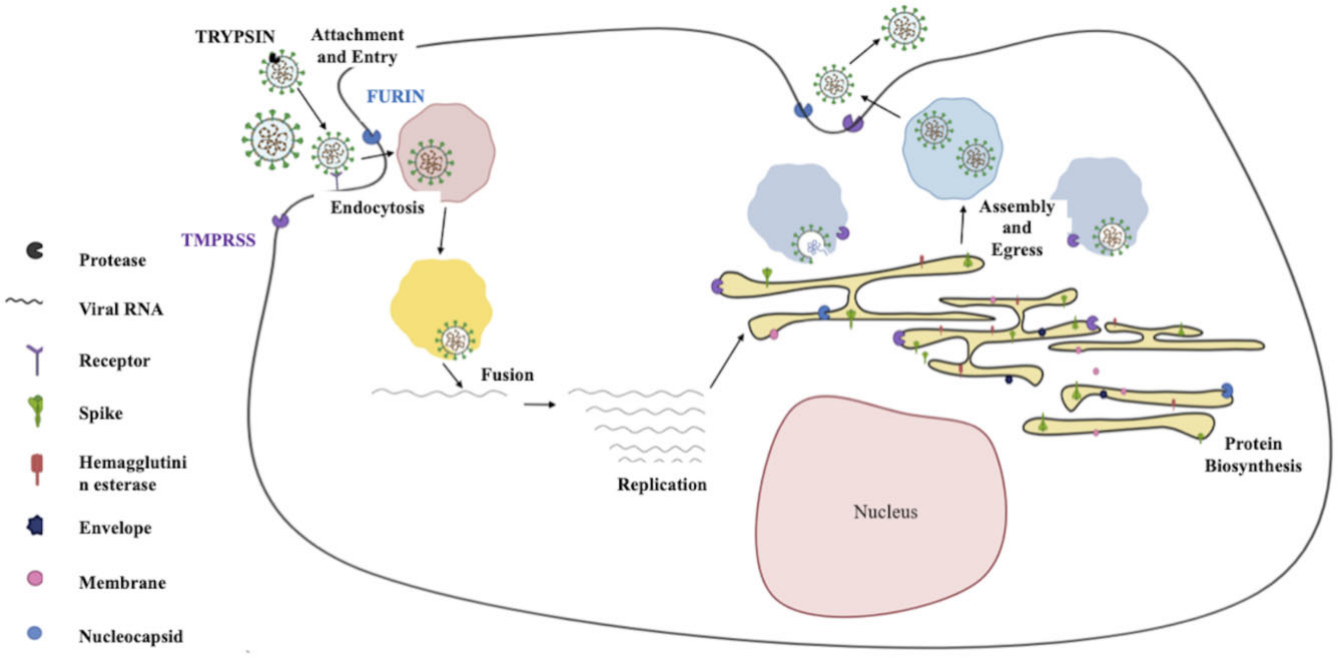
Schematic representation of SARS-CoV-2 infection and propagation in a host cell.

The structural protein, such as spike (S) is a transmembrane trimetric glycoprotein consisting of the S1 subunit and S2 subunit, on the surface of CoVs, which determines their diversity and host tropism. The S1 subunit is responsible for binding host cell receptors, and the S2 subunit is responsible for fusion with cellular membranes. The functional receptor for SARS-CoV was recognized as zinc peptidase ACE2 [40]. It has also been found by structural and functional analyses that spike for SARS-CoV-2 also destined to ACE2 expression [41, 42]. The binding of SARS-CoV-2 to the host cellular membrane leads to the activation of spike protein by protease cleavage at two distinct sites in a sequence. The cleavage at the S1/S2 site for priming, and for activation cleavage at S2 site, position in line to the fusion protein in S2 subunit, causing irreversible and conformational transitions [43, 44]. These S1 and S2 subunits remain noncovalently bound. However, the distal S1 subunit provides stability at perfusion state to the membrane-anchored S2 subunit [42]. The spike of CoV is unusual among other viruses as it can be cleaved and activated by different proteases [43] mainly coming from four different stages of the virus infection cycle (Fig. 3).

The unique characteristic of SARS-CoV-2 among all CoVs is the existence of furin protein cleavage site at S1/S2 subunit site. In contrast to the SARS-CoV spike, S1/S2 site is subjected to cleavage during the biosynthesis of SARS-CoV-2 [42]. Although S1/S2 site is also cleaved by other proteases such as transmembrane protease serine 2 (TMPRSS2), cathepsin L and cathepsin B [44, 45], the expression of furin ubiquitously makes this virus highly pathogenic. ACE2 is the receptor used by both SARS and SARS-CoV-2. Apart from ACE2 in humans, SARS is also compatible with ACE-2 in cat, rhesus monkeys, rabbit, dog and pig. The receptor usage of SARS-CoV-2 is broad, which implies that it has a broad spectrum of host range [46]. ACE2 has a diverse efficiency in various animals, which might highlight differences in susceptibilities to COVID-19 infection. The S protein in SARS-CoV-2 requires proteolytic processing for the endocytic route to be activated. Host proteases such as TMPRSS2, Furin and Cathepsin L are observed to be a part of S protein cleavage and activation of SARS-CoV-2 entry [47] (Fig. 3). The data from single-cell RNA sequencing depicted that TMPRSS2 has surprisingly high expression in several tissues. TMPRSS2 is also observed to be co-expressed along with ACE2 in lungs, epithelial cells of the nasal cavity and branches of bronchi. This also explains tissue tropism of SARS-CoV-2 to some extent (cell entry mechanisms of SARS-CoV-2 |).

The SARS-CoV-2 virus can infect nasal cells, pneumocytes and bronchial epithelial cells during the nascent stages of infection, by the process of binding of S protein to the ACE2 receptor, which is facilitated by the help of serine protease [3]. TMPRSS2 cleaves ACE2 and activates the spike protein of SARS-CoV-2 and hence is responsible for promoting viral uptake [3]. Both ACE2 and TMPRSS2 receptors are expressed majorly in alveolar epithelial type II cells. In case of severe disease, the chances of lymphopenia increase where SARS-CoV-2 infects and kills T cells and hence the count falls drastically less than 1000. Moreover, inflammatory viral response disrupts the mechanism of lymphopoiesis and encourages apoptosis of lymphocytes [48]. Entry assays of the SARS-CoV-2 virus have shown that cathepsin L, TMPRSS and furin have combined effects on viral entry activation.

While most viruses are known to mutate and change over time, several of these causes can trigger changes in virology that causes differences in infectivity, severity of disease and influence the efficacy of therapeutics. Throughout COVID-19 pandemic, diverse variants emerged worldwide. To monitor the emerging variants, WHO classified SARS-CoV-2 variants into three classes: variants under monitoring, variants of interests and variants of concerns (VOCs). Alpha (B.1.1.7), Beta (B.1.351), Gamma (P.1), Delta (B.1.617.2) and Omicron (B.1.1.529) are major VOCs emanating globally. According to WHO report, the first Omicron-infected case was reported on 9 November 2021. Since the emergence of the Omicron (B.1.1.529) variant, it has spread to different countries and continues to be the reason for COVID-19 infections. Sequence analysis of the Omicron variant ascertained several nonsynonymous mutations in the spike region that consequently leads to escalated transmissibility and unfavorable clinical outcomes. Understanding related emergent variants is still advancing which can be further employed for developing better strategies to combat the ongoing COVID-19 pandemic.

## Clinical manifestations of COVID-19

**Figure 4.**
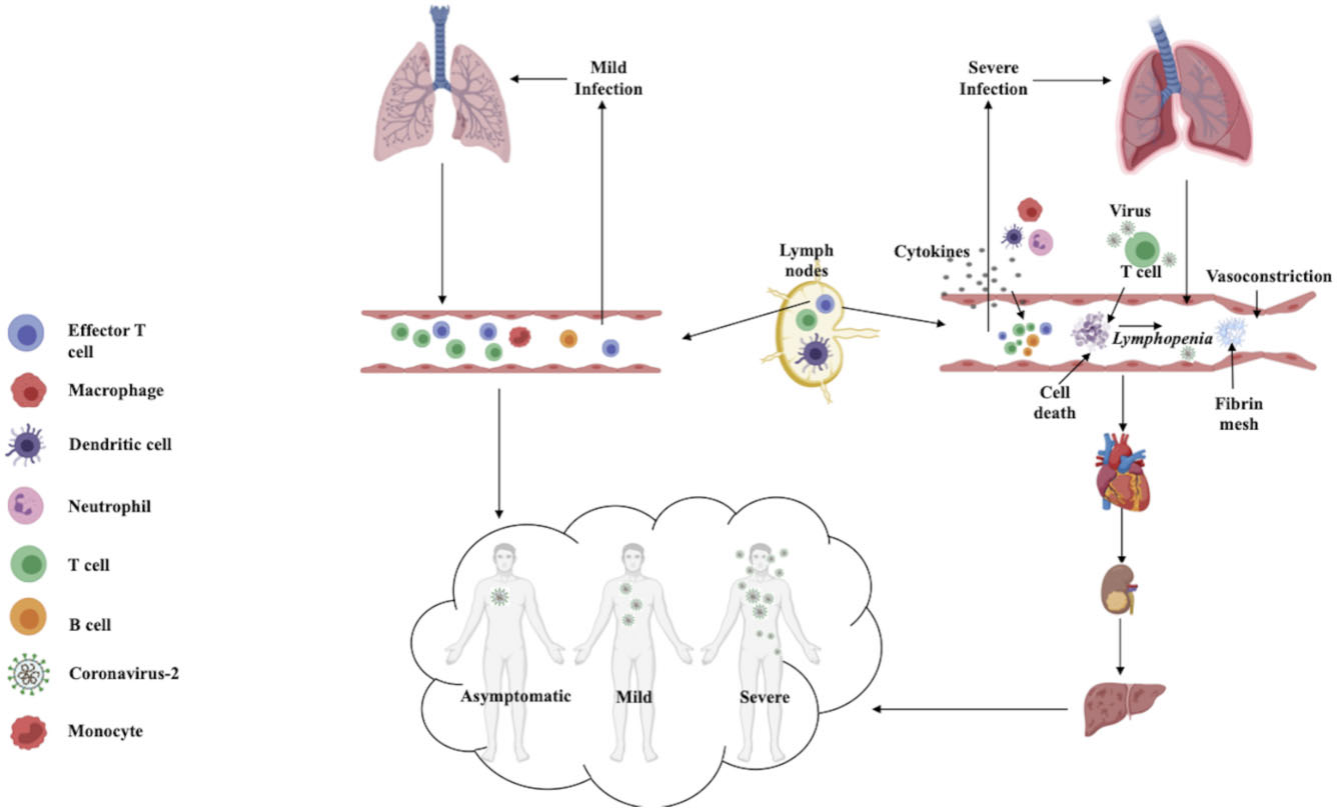
Heterogeneous clinical manifestations of COVID-19 spanning from mild infection to severe inflammatory damage.

Information regarding the existing COVID-19 pandemic is streamlining continuously. However, clinical symptoms of SARS-CoV-2 infections spanning from asymptomatic infection to severe death-instigating disease remain consistent with preliminary reports (Fig. 4) [49, 50]. The initial description of clinical symptoms of COVID-19 (formerly called 2019-nCoV) was reported for 41 hospitalized patients in Wuhan with laboratory-confirmed SARS-CoV-2 infection. Clinical manifestations observed in patients infected with nCoV highly resembled SARS-CoV and MERS-CoV infections [51, 52]. The majority of patients at the onset of the disease displayed fever, cough, fatigue, lymphopenia, headache and abnormal chest computed tomography (CT) representations with bilateral involvement. Symptoms such as leucopenia, sputum production, headache, hemoptysis, vomiting, nausea and diarrhea were exhibited by few patients. In due course of 8 days, over half of the patients experienced dyspnea or shortness of breath (SOB). Apart from pneumonia, COVID-19-associated complications such as acute respiratory distress syndrome (ARDS), RNAaemia, acute cardiac damage, multiorgan failure (MOF) and secondary infections were frequently observed. In critical patients, invasive mechanical ventilation and oxygen supplementation were indispensable [49].

Similar to SARS-CoV, the median incubation period for SARS-CoV-2 was estimated to be around 4 to 5 days [53]. Epidemiological investigations established that symptomatic individuals can develop symptoms up to 14 days after contracting an infection [54]. Supplementary information attained from the descriptive analysis of 99 laboratory-confirmed COVID-19 patients reinforced prior clinical observations. Almost half of the infected patients had chronic diseases like diabetes, cardiovascular and cerebrovascular disorders. Apart from common symptoms, muscle ache, confusion and chest pain were reported in some cases. Several patients underwent organ function damage that included acute respiratory injury, acute renal injury, septic shock and ventilator-associated pneumonia. Escalated cytokine levels in serum and cytokine storm-induced clinical severity were detected (Fig. 4). Lower platelet levels, raise in procalcitonin, serum ferritin and C-reactive protein (CRP) were detected in most patients. Additionally, liver function aberrations with elevation in alanine aminotransferase (ALT) or aspartate aminotransferase (AST) levels, deviant myocardial zymogram with irregular levels of lactate dehydrogenase and creatine kinase were frequently observed in patients [49]. Concurring observations from a report of 72 314 COVID-19 patients in China further validated heterogeneous clinical manifestations, encompassing 81% mild cases (outlined as no pneumonia or minor pneumonia), 14% severe cases [outlined as respiratory frequency ≥30 breaths/min, oxygen saturation (SpO_2_) ≤93%, a ratio of arterial partial pressure of oxygen to fraction of inspired oxygen (PaO_2_/FiO_2_) 50% within 24–48 h] and 5% critical cases (outlined as pulmonary failure, septic shock and/or multiorgan dysfunction) [55].

Patients in the initial stages of the pandemic were mostly older males, now it is established that individuals of all age categories are at risk but likelihood of severe COVID-19 is more in individuals with impaired immune responses, underlying diseased state and aging ≥60 years [56]. While SARS-CoV-2 infection primarily triggers pulmonary impairment, several reports reveal detrimental influence on other organs; for instance, COVID-19-associated complications in the heart [57], brain [58], liver [59] and kidney [60]. Extreme inflammation in critical COVID-19 patients can also provoke thromboembolic incidences [61]. Various reports of patients with persistent symptoms after recovering from acute COVID-19 are emerging. So far, there is no definite terminology and categorization, and long-term symptoms associated with COVID-19 are referred to as long COVID [62]. Reported persistent symptoms comprise fatigue, chest pain, SOB and psychological concerns after hospital discharge [63]. Till now the collective term used to describe several clinical features persistent after COVID-19 recovery remains long COVID. Persistent symptoms post-infection such as fatigue, cough, breathlessness, chest tightness and palpitations can be associated with organ damage or post-viral syndrome.

To improvise clinical management of emerging COVID-19-associated complications, more progress in research and observational cohort analyses is a prerequisite for better insight into post-acute COVID-19 sequelae.

## COVID-19 diagnostics

From initial hospitalizations, the detection of SARS-CoV-2 infection has been the foundation of monitoring the COVID-19 outbreak. Prompt diagnosis and immediate isolation are vital to limit SARS-CoV-2 transmission and save lives. Clinical manifestations of SARS-CoV-2 infection span from asymptomatic infection to severe death-instigating disease [49, 50]. In addition to heterogeneous observable symptoms that range from flu-like conditions to SARS [49], auxiliary lung impairment and a spectrum of inflammatory impairment in various organs have also been reported in COVID-19 patients [64]. Hence, timely and accurate diagnosis of SARS-CoV-2 infection is crucial to restrict transmission and to initiate a suitable therapeutic approach that can substantially improve outcomes and prevent mortality. Diverse testing approaches are deployed in an attempt to validate or dismiss COVID-19 infection as soon as an individual puts up with the WHO case definition for speculated or probable COVID-19. Routine thermal screening is implemented in high-throughput zones to detect people with fever (elevated normal body temperature) since it is a common symptom of COVID-19 [49]. CT an established, noninvasive technique was employed for assessing early hospitalizations with fatal pneumonia. All patients had peculiar bilateral involvement, evident in chest CT images [49]. Therefore, during the shortage of SARS-CoV-2-specific diagnostics, CT scans were employed in Wuhan to detect lung-related hallmark COVID-19 features [65]. Currently, CT scans are advisable for prognosis and detection of COVID-19-associated complications as an adjunct diagnostic assessment (use of chest imaging in COVID-19).

Based on the clinical and epidemiological speculations, individuals can be distinctively diagnosed for SARS-CoV-2, by either detecting viral components (RNA or antigens) or immune response specific to SARS-CoV-2. Detection of viral RNA or antigens is prioritized for early diagnosis, and antibody detection is employed for seroprevalence and epidemiological investigations. Until now, innumerable COVID-19 tests have been performed worldwide, and numerous reports have evaluated their performance, precision and consistency for SARS-CoV2 detection, which will be reviewed here.

### Molecular detection of SARS-CoV-2

Detection of unique segments of viral genetic material is the gold standard for the diagnosis of SARS-CoV-2. Prompt identification and sequencing of nCoV SARS-CoV-2 enabled rapid development of molecular diagnostics for COVID-19 detection [66]. According to WHO recommendation upon speculation, SARS-CoV-2 infections must be routinely diagnosed utilizing nucleic acid amplification test (NAAT) approaches for early diagnosis. Among all, reverse transcription–polymerase chain reaction (RT–PCR) is the most commonly used technique to detect characteristic viral RNA sequences in accessible patient clinical samples like nasopharyngeal swabs, nasal aspirates, sputum, BAL, etc. by using SARS-CoV-2-specific primers. Established targets for COVID-19 detection comprise conserved regions of *E, N, S, RdRp* and *ORF1a/b* genes from the SARS-CoV-2 genome [66]. Preferably, NAAT assays must aim at a minimum of two self-sufficient targets on the SARS-CoV-2 genome; however, in case of urgency, unambiguous unique targets can be employed in regions with prevalent SARS-CoV-2 transmission. Erroneous RT–PCR results are primarily associated with disparity in sensitivity due to sample collection and handling [49, 67] and accuracy differing with viral load [68]. Till now, more than 200 molecular diagnostics have received emergency use authorization (EUA) from the United States Food and Drug Administration (US FDA) (Health 2022a). One of which is Xpert Xpress with high sensitivity and 95% specificity [69], and quantitative RT–PCR is predominantly employed for case identification but have limitations. To overcome these limitations, like inaccessibility to reagents, equipment and logjams around the globe, several platforms were adapted to ease SARS-CoV-2 diagnostic. RT–PCR from diverse samples can be performed on an automated GeneXpert instrument, which integrates sample preparation, RNA extraction, amplification and detection improving the sensitivity and specificity (Xpert^®^ Xpress SARS-CoV-2 has received FDA Emergency Use Authorization). Another NAAT approach is RT–loop-mediated isothermal amplification (LAMP) that is accessible commercially as molecular point of care (POC) tests [70]. Since, comparable sensitivities as RT–PCR can be attained instantly at a single temperature without requiring specific laboratory equipment [70]. An alternate approach based on Cas13a ribonuclease-mediated RNA-sensing strategy, Specific High Sensitivity Enzymatic Reporter UnLOCKing (SHERLOCK) [71], has also been refined for SARS-CoV-2 testing. SHERLOCK testing in one pot COVID (STOPCovid) is a one-step fluorescence-based lateral flow assay that can detect infectious agents in samples and provide results within an hour as a single or double line on a paper strip [72]. Equipment-free prototype DNA Endonuclease-Targeted CRISPR Trans Reporter (DETECTR) developed by Mammoth Biosciences executes RT–LAMP and Cas12-mediated cleavage simultaneously for detection of SARS-CoV-2 RNA in samples to deliver results in <2 h [73, 74]. Finally, the FDA’s historic EUA approval of the first NGS test for COVID-19 testing to manage with testing demands highlights progression in molecular diagnostics. Along with COVID-19 diagnosis, sequencing can additionally monitor mutations in the viral genome which cause mismatches with primers and reduction in NAAT sensitivity. Systems are customized to process 1536 to 3072 clinical samples and deliver results in 12 h. The Illumina COVIDSeq test can qualitatively detect SARS-CoV-2 viral RNA in respiratory samples by amplicon or targeted RNA sequencing with 97% specificity and 98% sensitivity. Since, the majority of molecular diagnostics are limited to laboratories as they necessitate infrastructure, trained personnel or apparatus which can be challenging to access in many circumstances. Reliable immunoassays that can be employed at decentralized settings to provide rapid results are used to diagnose acute infections.

### Immunological detection of SARS-CoV-2

Immunoassays detect SARS-CoV-2 infection by either directly detecting viral protein components (antigens) or virus-specific host immune response (antibodies) in patient samples. Over 600 immunoassays are commercially available for COVID-19 diagnosis [95]. Immunoassays in the form of easy-to-use rapid diagnostic tests (RDTs) are used at or near the POC to ascertain, validate or investigate patient’s clinical condition without necessitating laboratory equipment. Most of the RDTs are lateral flow immunoassays (LFIs), designed on principles of enzyme-linked immunosorbent assay (ELISA) and chemiluminescence immunoassay (CLIA) to deliver results immediately and are relatively less expensive than NAAT. Most approved RDTs utilize respiratory samples; however, approval of rapid saliva test by the FDA (SalivaDirect^TM^ < SalivaDirect^TM^) has unlocked the door for alternate samples for proficient testing. Since the SARS-CoV-2 virus replicates in host throughout active infection and sheds antigens, detection of antigens in respiratory tract specimens is utilized to diagnose active infection within the first week following the onset of clinical symptoms. Ag-RDTs detect viral antigens, like nucleocapsid, viral spike S protein, which are abundantly circulating in respiratory samples. Ag-RDTs can be implemented for early diagnosis as viral load is highest in pre-symptomatic phase (1–3 days prior to the onset of symptoms) and early-symptomatic phase (within the first week of infection) of COVID-19 [75, 76]. Eventually, as the infection clears and patient recovers, Ag-RDT results turn negative. However, a negative result does not rule out infection and supplementary test is requisite to confirm the result. The accuracy of Ag-RDTs varies according to the concentration of antigen in the sample, viral load in patients at that particular stage of infection and many additional variables. Easy to use, low-cost and high specificity (97–99%) make them best suited for use as a first-line test and at the POC to determine, verify or confirm the patient’s clinical condition. Although, if judged alongside RT–PCR, since there is no amplification of the target that is detected, Ag-RDTs can yield variable and inadequate sensitivity. Hence, owing to relatively higher risk of false-negative results compared to RT–PCR, the WHO guidelines recommend rapid antigen tests for COVID-19 infection detection with at least ≥80% sensitivity and ≥97% specificity, only when NAT is unavailable in a setting or when medical practicality necessitates shortening of turnaround interval (antigen detection in the diagnosis of SARS-CoV-2 infection using rapid immunoassays). The alternative immunodetection approach is serological detection of antibodies elicited by the host in response to SARS-CoV-2, which persists during active infection and even after the virus is no longer detectable. The FDA has issued EUA to more than 70 SARS-CoV-2 serology/antibody tests that detect the presence of binding antibodies (Health 2022b, https://www.who.int/campaigns/world-health-day/2022) to determine past COVID-19 infection. However, binding antibodies are not as significant as neutralization antibodies which can obstruct cellular infiltration and replication of SARS-CoV-2 [77]. Virus neutralization assays are regarded as the gold standard test for confirming the existence of competent antibodies but due to the indispensable necessity of BSL-3 culture amenities and trained personnel, it is not used for routine testing. Therefore, principles of ELISA and CLIA are harnessed in serological assays to identify COVID-19 infection in patients for 15–21 days after the onset of symptoms [78] by assessing binding antibodies [total immunoglobulins (Ig), IgG, IgM and/or IgA in different arrangements]. Blocking ELISA-based qualitative assay that imitates virus neutralization assay and sense neutralization antibodies against SARS-CoV-2 received EUA to identify individuals with prior COVID-19 infection. However, the WHO does not recommend antibody detection for determining active COVID-19. Since generation of antibodies takes time, antibody detection assay is more suitable for seroprevalence surveys than primary testing. Hence, it majorly employed post-COVID-19 as a restraint strategy.

Irrespective of the approach employed for diagnosis, results must be elucidated while keeping the precision of test and associated risk factors into consideration. It is critical to enforce an innovative outlook in diagnostics technology and multiple established tests to sustain inflating demands in all circumstances. Now focus should be on harnessing pioneering technologies and strategies to improve sensitivity and specificity with minimum processing time at an economical price. Diagnostic lessons learned during the first and second waves of the COVID-19 pandemic should be used to help cope with the next wave. Ending the pandemic will involve application of diagnostic testing in high volumes and the rapid results to aid implementation of appropriate therapy and prevent further spread.

## Immune response to SARS-CoV-2

**Figure 5.**
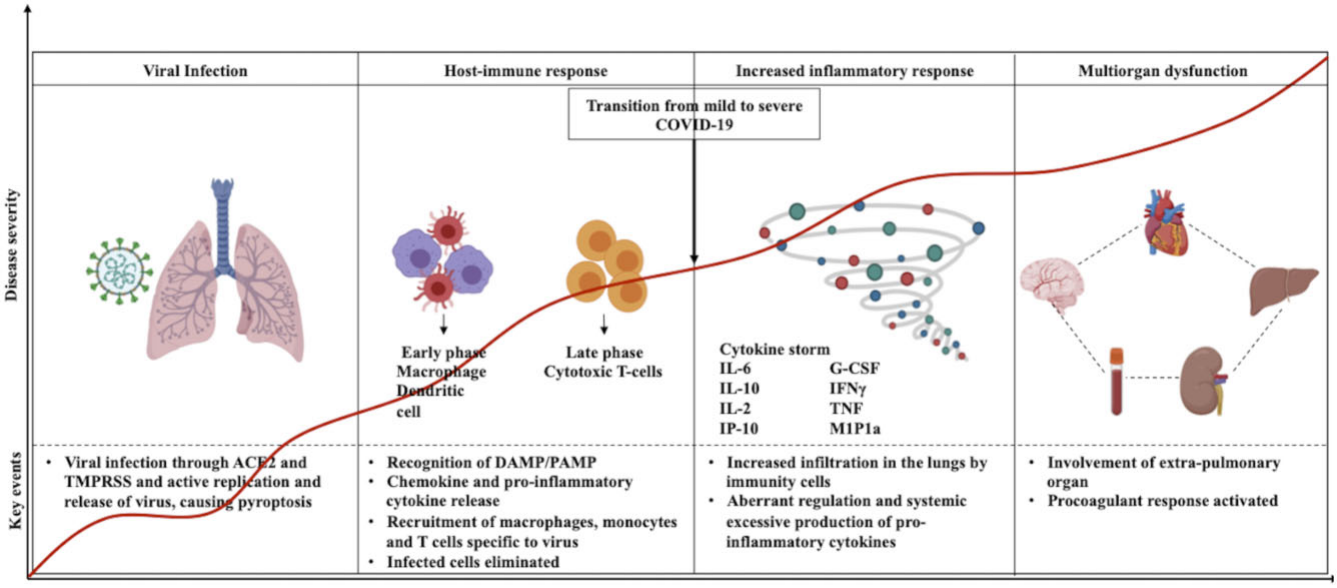
Immune responses and immunopathogenesis associated with SARS-CoV-2 infection.

The immune system of most individuals suffering from COVID-19 can self-sufficiently limit and exterminate SARS-CoV-2 infection. However, the endeavor to eliminate the virus can culminate into escalated inflammatory responses that direct later phases of COVID-19 disease and cause immunopathogenesis. The goal is to strike a balance for the resolution of infection exclusive of detrimental inflammation. Till now we have attained substantial knowledge regarding innate and adaptive immune responses against SARS-CoV-2 which will be reviewed in this section (Fig. 5).

### Innate immune responses to SARS-CoV-2

The innate immune system acts as the first line of defense in confronting SARS-CoV-2 infections. Subsequent to SARS-CoV-2 entry in host, the virus encounters pulmonary epithelium. The SARS-CoV-2 formerly infects pulmonary epithelial cells and further propagates to infect diverse respiratory cells. Host innate immune response is drawn out as soon as the virus infects the host cells [79]. Components of innate immunity recognize pathogen-associated molecular patterns and danger-associated molecular patterns associated with SARS-CoV-2. Viral RNA is recognized by distinct pattern recognition receptors (PRRs) such as Toll-like receptors (TLRs), nucleotide-binding oligomerization domain-like receptor family proteins [80] and retinoic acid-inducible gene I (RIG-I)-like receptors [81]. Primary constituents of the virus are detected by TLRs 3, 7 and 8, which lead to escalation in type I interferons (IFNs) production [82]. This triggers the expression of IFN-stimulated genes by neighboring infected cells, which brings about the release of proinflammatory cytokines, such as interleukin-6 (IL-6) and tumor necrosis factor (TNF) [83]. Furthermore, PRRs can assemble to form inflammasomes in SARS-CoV-2-infected pulmonary pneumocytes to instigate membrane pore generation, escalation in cytokine production that results in pyroptotic cell death and exhibition of DAMPs, such as adenosine triphosphate (ATP), nucleic acids and ASC oligomers. Alveolar macrophages can recognize these signals and elicit an immune response against the virus by generating pro-inflammatory cytokines and chemokines [84]. This prompts an antiviral state to combat SARS-CoV-2 infection. Cyclic GMP-AMP synthase-stimulator of interferon genes (cGAS–STING) pathway is an important regulator of peculiar Type 1 IFN responses to COVID-19 [85]. Pharmacological inhibition of STING in mice reduces severe lung inflammation and improves disease outcomes that suggested a relevant basis for the development of host-directed therapeutics [86].

However, prior information regarding SARS-CoV and MERS-CoV infers that antiviral response of IFN is altered by nonstructural viral components. This influences the efficiency of innate immune response and intensifies pro-inflammatory cytokines [87]. Similarly, accessory protein Orf6 of SARS-CoV-2 can restrain IFN responses in infected cells [88]. Restrained antiviral defenses of innate immunity combined with animated inflammatory responses largely determine the consequence of COVID-19 [89].

### Adaptive immune responses to SARS-CoV-2

Inefficacy of innate immune responses to eradicate SARS-CoV-2 triggers adaptive immune responses. SARS-CoV-2 infection in alveolar cells of lungs leads to infiltration and activation of T and B lymphocytes [90]. Resembling other CoV infections, humoral immune response against SARS-CoV-2 comprises generation of efficient IgG and IgM antibodies. At first, B lymphocytes exhibit prompt response against extremely immunogenic N protein, which leads to the emergence of N protein-specific nABs during the initial phase of infection [91]. Eventually, S protein-specific antibodies can be detected during the later phase of the disease. The SARS-CoV-2-specific antibodies can be detected at distinct timings; at first, IgM and IgA can be detected 5 days after the appearance of symptoms [92]. Afterward, IgG antibodies can be detected at Day 14 and prevail for an extended period [93]. In a cohort of healthcare personnel, the existence of anti-S IgG nABs stably for almost 5 months after SARS-CoV-2 infection was linked to invulnerability from reinfections [94]. In contrast, elevated levels of anti-N IgM and IgG were detected in severe COVID-19 patients [95]. This implies that apart from nABs that have a defensive function, several non-nABs exist and are involved in immune evasion by SARS-CoV-2. Antibodies existent from prior SARS-CoV infection can facilitate entry of SARS-CoV-2 in cells expressing Fc receptors such as monocytes, macrophages and B lymphocytes [90]. Fc receptor-mediated viral entry in macrophages by antibody-dependent enhancement does not initiate viral replication and dissemination. This progression stimulates myeloid cells to augment inflammation and tissue damage [95]. Moreover, autoantibodies were discovered in severe COVID-19 cases and patients with persistent COVID-19 symptoms (long COVID) [96]. The manifestation of autoantibodies targeting tissue-specific antigens might instigate antibody-mediated organ damage in critical patients [97].

Infiltration of lymphocytes from blood into the site of infection is directed by elevated pro-inflammatory cytokines and chemokines such as IL-6, IFNγ, MCP1 and IP-10 in the blood of COVID-19 patients [84]. This also explains lymphopenia and larger neutrophil-to-lymphocyte ratio monitored in the majority of COVID-19 patients [98]. T lymphocytes are recruited by infected macrophages to exterminate SARS-CoV-2-infected cells and for resolution of infection. Pro-inflammatory cytokines polarize CD4^+^ T cells into T helper 1 (Th1) cells that secrete Granulosite-macrophage colony-stimulating factor (GM–CSF) and stimulate monocytes to release IL-6. An enlarged subgroup of CD14+IL-1β^+^ monocytes can elevate IL-1β in COVID-19 patients [99]. Furthermore, the Th17 response in COVID-19 patients [100] instigates pro-inflammatory cytokine IL-17, which employs monocytes and neutrophils to pulmonary circulation and initiates a surge of surplus cytokines such as IL-1β and IL-6 [100]. Heightened immune response by a plethora of immune cells leads to dysfunctional immune response and augmented inflammation [101]. In the majority of COVID-19 cases, immune cells proficiently clear viral burden, following which immune response regresses and patients convalesce. But in some patients, cytokine storm is elicited which results in inflammation of the lung. Cytokine storm and upsurge of cytokines such as IL-2, IL-7, IL-10, G-CSF, IP-10, MCP1, MIP1α and TNF are frequently observed in severe SARS-CoV-2 infections. Intensifying IL-6 levels in critical patients is a risk factor for mortality [101].

Unrestricted permeation of immune cells can result in pulmonary impairment due to immense emission of proteases, oxidative stress and damage associated with propagation of the virus. This can trigger diffuse alveolar injury comprising pulmonary edema, desquamation of alveolar cells and hyaline membrane formation [102]. These impediments can hamper gaseous exchange in alveolar regions of the lung, which gives rise to breathing concerns and low levels of oxygen in the blood. Additional to tissue injury in pulmonary region, dysregulated TNF levels can instigate septic shock and MOF in COVID-19 patients [103].

Therefore, restricting dysregulated immune response is as important as targeting SARS-CoV-2 for robust clinical trajectories. Extensive knowledge regarding determinants of immune dysfunction is paramount to establish suitable immunomodulatory treatment for SARS-CoV-2 infections.

## Therapeutics of COVID-19

### Antiviral therapy

Several clinical manifestations of COVID-19 are instigated by replication of SARS-CoV-2. Antiviral therapies are aimed to control multiplication and establishment of SARS-CoV-2 in initial course of COVID-19. Obstructing replication phase initially can circumvent the advancement of disease to disorderly inflammatory later phases [104]. Hence, stages of viral lifecycle are prospective targets for therapeutics. Antiviral drugs function by blocking viral entry through ACE2 receptor and TMPRSS2; critical processes such as viral membrane fusion and endocytosis; or action of non-structural proteins such as 3CLpro and RdRp. Analogous to other viral infections, to cure COVID-19, combination of synchronous drugs targeting distinct viral processes is required for prompt SARS-CoV-2 clearance.

**Figure 6.**
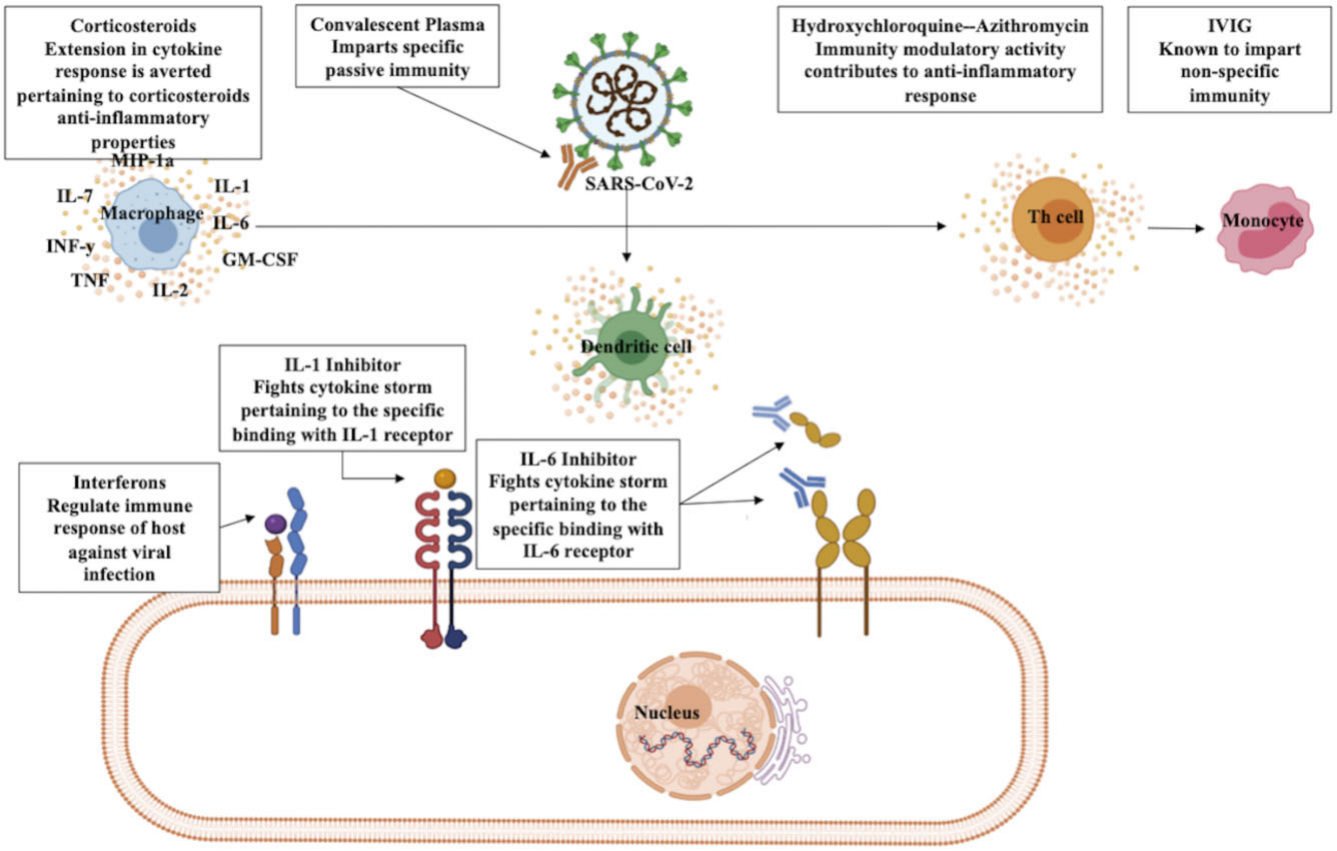
Potential therapeutic approaches for management of COVID-19.

Information regarding antiviral medications investigated to treat mild-to-moderate, severe and critical COVID-19 patients is summarized in this section (Fig. 6).

Hydroxychloroquine, which is in general prescribed for treating malaria and in particular autoimmune disorders (Drugs@FDA: FDA-approved drugs) in the initial course of the pandemic, was used for the treatment of COVID-19. Owing to its immunomodulatory potential and ability to prevent the transport of SARS-CoV-2 from early endosomes to endolysosomes, medication was evaluated for COVID-19 treatment. Despite affirmative *in vitro* antiviral potential, in the rhesus macaque model, viral load reduction and efficacious outcomes were not attained with hydroxychloroquine treatment [105]. Furthermore, assessment in observational studies and randomized trials confirmed inefficacy and adverse consequences of hydroxychloroquine treatment for COVID-19.

Remdesivir, another medication, which was earlier identified as GS-5734 is an intravenous monophosphate prodrug with antiviral activity against single-stranded RNA viruses. It is an ATP analog that gets metabolized and is converted into an active form, C-adenosine nucleoside triphosphate analog. Broad-spectrum antiviral activity of remdesivir is attributable to its ability to inhibit viral RdRp and replication of the viral genome. Since it exhibited *in vitro* activity against SARS-CoV and MERS-CoV [106], antiviral activity was further evaluated for SARS-CoV-2 [107]. In several clinical trials, remdesivir treatment led to better health outcomes and reduction in clinical recovery in hospitalized COVID-19 patients [108, 109]. Veklury (remdesivir) is currently the only FDA-approved drug for COVID-19-treating hospitalized adults and pediatric patients (aged ≥12 years and weighing ≥40 kg). Moreover, it has received EUA from FDA for treating hospitalized pediatric patients weighing 3.5 kg to <40 kg or aged <12 years and weighing ≥3.5 kg. However, it can induce nausea, hypersensitivity and escalate transaminase levels.

Favipiravir (FabiFlu), an established antiviral drug, which serves as a prodrug approved to cure resistant influenza strains. It is a modified pyrazine analog that inhibits viral RdRp and prohibits viral transcription and replication. Since it acts proficiently for diverse influenza strains [110], the effectiveness of favipiravir was examined against SARS-CoV-2. In agreement with former investigations, favipiravir was found to facilitate clinical recovery and possibly will be favorable in the treatment of mild-and-moderate COVID-19 patients in phase 3 clinical trial.

Ivermectin, another potential medicine, is an FDA-approved semi-synthetic broad-spectrum anti-helminthic drug [111, 112]. It can restrict viral infections by binding to host importin alpha/beta-1 (IMPα/β1) nuclear transport proteins [113]. IMPα/β1 transporters are involved in SARS-CoV infection and impact host cellular division. Hence, this inhibitory activity of ivermectin was proposed against SARS-CoV-2 infections. Preliminary *in vitro* experiments on Vero-hSLAM cells demonstrated reduction in viral RNA on ivermectin treatment [114]. However, this was achieved by administering high doses that can cause adverse effects [115]. Clinical trials have evaluated the use of ivermectin in COVID-19 patients but due to limited information, the efficacy of ivermectin against SARS-CoV-2 is questionable.

### Anti-SARS-CoV-2 monoclonal antibodies

Passive immunization with monoclonal antibodies (mABs) targeted to recognize and neutralize virus is an efficient therapeutic approach for COVID-19 treatment. Prior knowledge regarding the kinetics of immune response subsequent to SARS-CoV infection indicated that in critical patients nABs crested earlier, diminished drastically and antibodies were constrained to only N protein, whereas in recovered patients, the peak antibody response was observed later with distinct isotypes against N as well as S viral protein [116]. Trimeric spike (S) glycoprotein is a major antigenic epitope on SARS-CoV and SARS-CoV-2 [92, 117]. Hence, targeting S glycoproteins that bind to ACE2 receptors and mediate entry into host cells can inhibit SARS-CoV-2 infection and obstruct viral pathogenesis [118]. Furthermore, elevation in SARS-CoV-2-specific nABs targeting S1, receptor-binding domain (RBD) and S2 region of S protein was detected in convalescent COVID-19 patients [119]. Neutralizing mABs with optimal affinity and specificity for significant regions of SARS-CoV-2 were derived from B lymphocytes of convalescent patients or humanized mice [120, 121]. Various pairs of efficacious noncompeting mABs-targeting RBD of the S protein were selected to counter the crisis of emergent mutant strains. Casirivimab (formerly known as REGN10933) and imdevimab (formerly known as REGN10987) were selected as they can simultaneously target different regions of RBD. Casirivimab binds at RBD from the top, masking binding site of ACE2, and binding site of imdevimab is positioned adjacent to RBD [122]. The FDA has issued EUA for combination therapy of casirivimab 1200 mg plus imdevimab 1200 mg to treat mild-to-moderate COVID-19 outpatients at risk of developing severe COVID-19. A combination of casirivimab and imdevimab can neutralize all SARS-CoV-2 mutants identified until now [123]. Similarly, the FDA has also issued EUA for REGEN-COV, that is combination of bamlanivimab 700 mg (also identified as LY-CoV555 and LY3819253) and etesevimab 1400 mg (also identified as LY-CoV016 and LY3832479) for treating mild-to-moderate COVID-19 outpatients. Both bamlanivimab and etesevimab bind at diverse but overlapping regions of RBD of S protein. This combination therapy reduced viral burden substantially at Day 11 in outpatients with mild-to-moderate SARS-CoV-2 infections [124]. Multiple clinical trials have evaluated the efficacy and safety of existing anti-SARS-CoV-2 mAB combinations [125]. Single infusion of mAB combination was sufficient to considerably reduce COVID-19-associated complications and mortality in outpatients. Most frequently monitored adverse effects included nausea, pruritis, pyrexia and hypersensitive reactions were observed in rare cases. Furthermore, evaluation of the SARS-CoV-2 variants and the impact of emerging mutations on the efficacy of anti-SARS-CoV-2 mAB combinations in population-based genomic surveillance is required.

### Immunomodulatory therapy

Immunomodulatory drugs can stimulate and/or suppress or regulate distinct components of the immune system to combat infections. Immunostimulators can be utilized to enhance the immune response against infectious diseases, although morbidity and mortality in critical COVID-19 cases are associated with hyperinflammation [99], and restraining the cytokine signaling using immunomodulatory agents may subside the hyperinflammation to some extent in the patients. We have described these therapies in detail in this section (Fig. 6).

#### Corticosteroids

Adjunct corticosteroids can modulate dysfunctional host immune responses. Corticosteroids were administered to limit inflammatory responses induced by SARS-CoV [126] and MERS-CoV [127]. Although they can prolong viral clearance [128, 129], yet usage of anti-inflammatory and immunosuppressive corticosteroids is preferred to alleviate systemic inflammation associated with severe COVID-19. From initial hospitalizations in Wuhan, a combined regimen for treating pneumonia included systemic corticosteroid therapy. A variable dose of methylprednisolone (40–120 mg per day) was administered to hospitalized COVID-19 patients to treat (ARDS). However, in phase IIb, randomized trial, course of 0.5 mg/kg methylprednisolone IV two times daily for 5 days was found ineffective to lower mortality in hospitalized COVID-19 patients [130]. Thus, further evaluation was required to validate the utility of corticosteroid therapy for treating COVID-19 patients. Results from the Randomised Evaluation of COVID-19 Therapy (RECOVERY) trial demonstrated that administering 6 mg dexamethasone every day for up to 10 days lowered the risk of progression to invasive mechanical ventilation and incidence of death in hospitalized COVID-19 patients. However, it was unclear whether receipt of dexamethasone was advantageous for patients not requiring respirational assistance [131]. In a retrospective cohort study, early, short-tenure corticosteroid therapy (oral prednisone or IV methylprednisolone) at low dosage was linked with deteriorating medical conditions and increased use of antibiotics [132]. Adverse consequences are frequently reported with the use of prednisone or methylprednisolone in COVID-19 patients with pulmonary co-infections [159]. The efficacy and safety of integrated corticosteroid therapy along with antiviral drugs for COVID-19 treatment are still uncertain. Better understanding of corticosteroids, selection of correct dosage regime and suitable clinical phase to initiate course is must for superior medical utilization for COVID-19 treatment.

#### Convalescent plasma therapy

Convalescent plasma therapy (CPT) involves transfusion of plasma from COVID-19 patients in the convalescent phase as a treatment for active SARS-CoV-2 infection in other patients. The basis of this immunotherapy is to establish passive immunity against SARS-CoV-2 by adoptive transfer of antibodies enriched plasma for better clinical outcomes in diseased individuals. During the Ebola virus disease outbreak, the usage of CPT was recommended by WHO as an investigative therapy to cope with outbreaks. Hyperimmune IV immunoglobulin isolated from convalescent patients’ plasma significantly reduced the viral burden and incidence of death in patients with severe influenza A (H1N1) [133]. In severe influenza and SARS-CoV infections, administration of CPT was found safe and was associated with quicker recovery and reduced mortality [134, 135]. Prior knowledge regarding SARS-CoV infections indicates that convalescent plasma comprises nABs targeted against viral S protein which can obstruct SARS-CoV entry via ACE2 receptors [136]. Since SARS-CoV-2 displays similar features like SARS-CoV, utility of CPT was evaluated for COVID-19 treatment. In a preliminary study, considerable nAB response was detected in plasma of convalescent COVID-19 patients [137]. A retrospective analysis concluded that transfusion of plasma with high anti-SARS-CoV-2 IgG antibody titer lowered the risk of death in hospitalized COVID-19 patients [138]. The FDA has announced EUA for administering high-titer COVID-19 convalescent plasma to treat hospitalized COVID-19 patients in the initial course of the disease or patients with compromised humoral immunity. Consultation with a transfusion medicine specialist is recommended prior to transfusion of convalescent plasma to inpatients with a history of severe allergic or anaphylactic transfusion reactions. In general, adverse reactions consequent to transfusion of COVID-19 convalescent plasma are uncommon. Nonetheless, recent evidence from the RECOVERY trial proclaims the ineffectiveness of high-titer convalescent plasma in improving clinical outcomes of hospitalized COVID-19 patients [139]. Further assessment is required to ascertain the safety and efficacy of convalescent plasma transfusion in SARS-CoV-2-infected patients.

#### IFNs

IFNs are released by the components of immune system to defend the host against pathogenic viruses. In prior investigations, timely inhibition of IFNα/β response was linked with dysregulated T-cell responses, inflammation and respiratory impairment during SARS-CoV and MERS-CoV infections [140, 141]. Furthermore, administering IFNα-2a in combination with antiviral therapy improved survival substantially in severe MERS-CoV patients [142]. However, due to inconsistent scientific data for SARS and MERS [143, 144], the utility of IFN usage for COVID-19 treatment was assessed by several research groups. *In vitro* experiments demonstrated potential efficacy and higher sensitivity of SARS-CoV-2 to IFN-α and IFN-β than SARS-CoV [145]. Serum profiles of COVID-19 patients presented depleted type I and type III IFN levels along with elevated pro-inflammatory cytokines [146]. Similarly, weakened IFN type I response in consort with persistent viral burden in blood was detected in critical COVID-19 cases [147]. Neutralizing IgG auto-antibodies against type I IFNs were detected in severe COVID-19 cases [148]. Moreover, mutations in type I IFN pathway were identified from sequencing data of COVID-19 patients with pneumonia [149]. In a clinical trial evaluating the impact of IFNβ-1a inhalation, reduction in breathlessness and better clinical outcomes were observed in hospitalized COVID-19 patients [150]. In Phase 2 trial, supplementing IFNβ-1b on alternating days along with double antiviral therapy was linked with improved medical state and briefer time to clear out viral load [151]. Similar outcomes were attained by administering nebulized IFNα-2b in a retrospective cohort study [152]. However, these outcomes remained questionable due to ill-defined treatment groups. IFN therapy can also instigate detrimental adverse effects. Existing information regarding the efficacy of early IFN treatment is inadequate to counsel either for or against treating SARS-CoV-2 infections.

#### IL-1 inhibitors

The immune response against SARS-CoV-2 can instigate dysfunctional immune response with an upsurge of pro-inflammatory cytokines. Elevated pro-inflammatory cytokine responses during the course of COVID-19 can cause injurious disease outcomes. In clinical analysis, it was observed that fever was the presenting symptom in 77.1% of the COVID-19 patients, which is largely driven by IL-1 [153]. Moreover, elevation in IL-1 levels has been strongly correlated with disease severity [154]. Induction of IL-1 during SARS-CoV-2 infection stimulates uncontrolled production of pro-inflammatory cytokines and triggers cytokine storm that causes lung damage and hampers respiratory capacity [155]. Hence, the utility of IL-1 inhibitors was assessed to control cytokine storm, inhibit organ failure and improve clinical outcomes.

Anakinra is a recombinant human IL-1 receptor antagonist. KINERET^®^ (anakinra) is approved by FDA for reducing symptoms, structural damage in rheumatoid arthritis patients and for treatment of cryopyrin-associated periodic syndromes. The only variation from native human IL-1Ra is additional methionine residue at amino-terminal in anakinra. Hence, it can proficiently obstruct pro-inflammatory response of both subtypes IL-1α and IL-1β by competitively inhibiting IL-1 from binding to IL-1 type I receptor. In prior trials, injection-site reactions such as erythema, joint pain was the commonly experienced side-effect (DailyMed—KINERET-anakinra injection, solution). Hence, based on the preliminary information, various clinical investigations analyzed utility of anakinra for treatment of SARS-CoV-2 infection. In multi-centric analysis, IL-1 receptor blockade by administering anakinra led to substantial survival benefits in sepsis patients with attributes of the macrophage activation syndrome (MAS) [156]. Henceforth, benefits of employing anakinra for restraining cytokine storm were evident from serological and medical improvement in adult MAS patients [157]. Based on these favorable results, utility of anakinra was further assessed in COVID-19 patients. Results of a cohort study elucidated reduction in necessity for invasive mechanical ventilation and mortality in severe COVID-19 cases treated with subcutaneous anakinra. This outcome was further validated when treatment with high dosage of anakinra was found safe and led to clinical improvement in 72% of the patients with COVID-19 and ARDS [158]. However, contradictory findings from a randomized Phase 2 trial indicate inefficiency of anakinra in treating patients with mild-to-moderate COVID-19 pneumonia [159]. Additional information from the ongoing clinical trials is requisite for certainty.

Apart from this, several groups are investigating the efficacy of Canakinumab, a biologic medicine approved for treatment of auto-inflammatory disorders under the trade name Ilaris^®^_._ It is a recombinant human IgG mAB that specifically binds and blocks IL-1β [160]. Based on promising reports of the previous clinical trials, multiple clinical studies are evaluating its repurposing to alleviate COVID-19-associated complications. However, a randomized, double-blind Phase III trial investigating efficiency of Canakinumab in hospitalized COVID-19 patients with pneumonia and cytokine release syndrome presented questionable outcomes. Repurposing of IL-1 inhibitor ARCALYST^*^®^*^ (rilonacept) which is recently approved for treatment of recurrent pericarditis for COVID-19 is also under investigation.

#### IL-6 inhibitors

Escalation in pro-inflammatory responses by an overpowering immune system directs detrimental pathological state. Various studies have reported elevation in IL-6 in COVID-19 patients [161, 162]. Furthermore, amplified IL-6 levels were associated with disease severity and mortality [163]. IL-6 is a pleiotropic cytokine produced by diverse cell types. IL-6-mediated downstream signaling can trigger vascular permeabilization, tissue damage and myocardial dysfunction [164]. Hence, inhibition of IL-6 was strategized to obstruct inflammatory responses and manage COVID-19-associated complications. The ongoing clinical trials are investigating potential of repurposing FDA-approved IL-6 inhibitors: siltuximab, sarilumab and tocilizumab for COVID-19 treatment.

ACTEMRA^®^ (tocilizumab) is a recombinant humanized anti-IL-6 receptor mAB, which efficiently inhibits IL‐6‐mediated immune signaling by binding to soluble and membrane-bound IL-6R. It is approved by FDA to treat various inflammatory and autoimmune illnesses. The utility of this well-established IL-6 inhibitor is presently under investigation to treat SARS-CoV-2 infection-associated adversities. Tocilizumab treatment reformed clinical manifestations and normalized body temperature, concentration of oxygen inhalation and oxygen saturation in severe and critical COVID-19 patients [165]. Certain clinical assessments generated contradictory outcomes which can be due to adequacies in patient enrolment measures [166, 167]. However, RECOVERY trial evaluating various therapies in hospitalized COVID-19 patients with hypoxia and systemic inflammation found tocilizumab to shorten the time of discharge and lessen mortality [168]. Likewise, in Randomised, Embedded, Multi-factorial Adaptive Platform Trial for Community-Acquired Pneumonia treatment with IL-6 receptor antagonist led to better survival in critically ailing COVID-19 patients on organ support admitted in ICUs [169]. Affirmative results from these trials incited further evaluation of tocilizumab for COVID-19 treatment. Nonetheless, treatment can lead to adverse effects such as dose-dependent elevation in liver enzyme levels and susceptibility to infections. Hence, the target should be to limit inflammation while averting secondary infections. Further analysis is requisite to distinctly establish patient population that will be benefited by incorporating tocilizumab in therapy.

Sarilumab is another recombinant humanized anti-IL-6 receptor monoclonal IgG1 antibody that has been approved by FDA to treat rheumatoid arthritis [170]. It proficiently binds membrane-bound and soluble IL-6 receptors and hinders downstream signaling [171]. Better survival rates in critically ailing COVID-19 patients were observed on treatment with IL-6 receptor antagonist [169]. However, in a multinational Phase 3 trial, sarilumab was found ineffective to treat hospitalized COVID-19 patients receiving supplemental oxygen [172]. Another class of IL-6 inhibitor under evaluation is IL-6 antagonist *SYLVANT*^®^ (*siltuximab*) which is a recombinant chimeric monoclonal IgG1-kappa antibody. It is approved by FDA for treating Castleman disease. It binds to human IL-6 and prevents it from binding to its receptor, obstructing IL-6 signaling-associated complications. In a retrospective study consequent to treatment with siltuximab in COVID-19 patients with pneumonia/ARDS, reduction and stabilization in serum CRP levels were observed. However, 33% of the patients exhibited clinical improvement, 24% of the patients’ clinical state deteriorated and one of which underwent a cerebrovascular incident and died. Additional information is needed regarding the safety and efficacy of siltuximab in patients with SARS-CoV-2 infection.

### IVIG therapy

IVIG is a product resulting from the serum combined from thousands of healthy donors. The major component of IVIG preparation is the serum IgG fraction consisting mainly of IgG1 and IgG2 categories [173]. Traces of IgA and IgM were also detected. At first, the logic behind its use was that it is being administered to patients with immunodeficiency due to hyperglobulinemia. Since then, it has been analyzed that IVIG exerts epistatic immunomodulating actions considering both innate and adaptive immunity; it has been used in various diseases such as neuromuscular, dermatologic and hematologic disorders [174]. However, The COVID-19 Treatment Guidelines Panel (the Panel) recommends against the use of SARS-CoV-2-specific IVIG for the treatment of COVID-19, except in a controlled clinical trial. In a controlled clinical study based on Latino patients suffering from COVID-19, it was observed that by the use of IVIG therapy, there was a significant reduction in respiratory failure and requirement of mechanical ventilation in COVID-19 patients.

## Vaccines for SARS-COV2

A vaccine is a biological preparation which can be administered through injection, inhalation or oral route that can stimulate immunity. It contains killed or attenuated microorganism, or one of its surface markers, or lab-generated altered form of its toxins. Various countries are in line to produce the most safe and efficacious vaccine, and COVID-19 vaccine candidates are being investigated by various technologies and platforms globally; these include live attenuated vaccines, nucleic acid vaccines, adenovirus-based vector vaccines, recombinant subunits vaccines, etc. After struggling through the pandemic and lockdown, now we have some promising options of vaccines already developed and approved for emergency use. Some of the categories of approved and efficacious vaccines, which are being given to the mass and showing promising results as well, are mentioned below with their dosage value efficacy rate and few other characteristic features.

### mRNA vaccines

#### Pfizer-bioNtech

New York-based Pfizer in collaboration with the BioNTech made history on 9 November 2020 by proving that their COVID-19 vaccine also known as Comirnaty, tozinameran or BNT162b2 has an efficacy rate of over 90%, when given in two doses, 3 weeks apart. The FDA conceded its first EUA for a vaccine on 11 December. The vaccine accustoms messenger RNA (mRNA) among the CoV proteins; S protein has been the most common preference. mRNA vaccines represent a promising substitute compared to the prevailing vaccines due to their high potency, ability for rapid development and cost-efficient production [175, 176]. In Phase 1 trials, the researchers discerned that Comirnaty triggered volunteers to produce antibodies as well as T cells against SARS-CoV-2; conversely, Phase 1/2 trials aiming the RBD of the S protein also proposed that the vaccine caused mild to restrained local and systemic symptoms in most vaccinators [177]. Other than that, this vaccine can only be kept at −15°C to −25°C which can become a setback when it comes to mass vaccination.

#### Moderna

On 18 December 2020, the FDA gave EUA for the vaccine made by the Boston-based company Moderna. The Moderna vaccine is the second vaccine approved by the FDA. It came a week after the Pfizer–BioNtech, it is again an mRNA-based vaccine that has to be given in two doses 4 weeks apart, which can be stored at 4°C (COVID-19 Vaccine Tracker: Latest Updates—*The New York Times*). The efficacy rate of vaccine is 94.1% although it is not yet known for how long the efficacy will last.

### Vector vaccines

#### Sputnik-V

It is also known as ‘gam-covid-vac’. The Gamaleya Research Institute, part of Russia’s Ministry of Health, has created this vaccine with an efficacy rate of 91.6%, when given two proper doses over the gap of 3 weeks. This vaccine is made from a combination of two adenoviruses called Ad5 and Ad26. Both of them have been tested as vaccines over the course of years. Phase 1/2 trail of recombinant adenovirus type 5 (rAd5) vector and a rAd26 vector, both carrying the S gene of SARS-CoV-2, demonstrated that the pre-existing immune response to the vectors rAd5 and rAd26 did not influence the titer of RBD-specific antibodies. Thus, make it as a good option to antagonize the negative impacts of immune response to vaccine vectors [178]. Sputnik-V has been approved and in use in Russia since November 2020.

Also, oddly enough, the Gamaleya Institute decided to collaborate with AstraZeneca that makes human adenoviruses vaccine, AZD1222 against COVID-19. The two combined their vaccines to study whether the mixture can enhance the efficacy of the AZD1222 vaccine; the trial began in February 2021.

#### AstraZeneca-Oxford (Covishield)

It is popularly known as Covishield in India and manufactured by the serum Institute of India, Pune. The efficacy rate of this vaccine is 82.4% for two doses separated over the course of 12 weeks, and it is a stable formula which can last up to 6 months in refrigeration. It consists of the nonreplicating simian adenovirus vector ChAdOx1 containing the full-length structural spike protein of SARS-CoV-2 developed by University of Oxford and AstraZeneca [179]. The researchers did not detect any severe side effects in the trials, but they noticed the increase of antibodies against the coronavirus as well as other immune defenses. The vaccine began Phase 2/3 trials in both the UK and India, and in addition to that AstraZeneca launched Phase 3 trials in Brazil, South Africa and the USA also. However, AstraZeneca’s COVID-19 vaccine has recently been flashed in news due to its temporary ban in Denmark, Irelands, Norway, Iceland and the Netherland due to the casualties believed to be caused by the vaccine in Denmark. Even though AstraZeneca has remarked on 14 March that there are no unswerving evidence that proposes fostered risk of blood clots due to the vaccine, a carefully appraised safety data of around 17 million people from European Union and UK, who took the AstraZeneca COVID-9 vaccine exhibited no evidence of such grave side effects, like pulmonary embolism or thrombocytopenia, of any defined age group or gender and on the basis of that vaccination among the mass has been resumed.

### Protein-based vaccines

#### Bektop-EpiVacCorona

On 14 October 2020, Russia gave regulatory approval to another vaccine named EpiVacCorona manufactured by a Russian research institute named ‘vektor institute’ and it consists of small portions of the viral protein, the vaccine banks on chemically integrated peptide antigens of SARS-CoV-2 proteins, linking to a carrier protein and adsorbed on an aluminum-containing adjuvant that is aluminum hydroxide, and it stimulates high immune responses due to their repetitive structures and are safer than other types of vaccines due to the absence of genetic material in them. It is being given in two doses, 3 weeks apart, and it has been said that it could be stored up to 2 years in stable refrigeration. The efficacy rate and Phase 3 trial results of this vaccine are yet to be published. Although a mass vaccination campaign with EpiVacCorona and Sputnik-V have already started in January 2021.

### Inactivated COVID vaccines

#### Bharat Biotech-Covaxin

Covaxin is the first indigenous COVID-19 vaccine by Hyderabad-based Bharat Biotech developed in collaboration with the Indian Council of Medical Research (ICMR) and National Institute of Virology (NIV). It is an inactivated COVID vaccine, which means it has inactivated or attenuated viruses that are made noninfectious via physical or chemical methods and comprise of multiple viral proteins for immune recognition, have secured expression of conformation-dependent antigenic epitopes and can be easily produced in large quantities [180]. It is a two-dose course given 4 weeks apart and stable at 2–8°C in refrigeration. Preclinical studies showed strong immunogenicity and great efficacy in animal models like hamster and nonhuman primates. In July 2020, the vaccine got approval from the Drug Controller General of India for first and second human-controlled clinical trials, 375 subjects in total were enrolled in Phase 1 study and showed magnificent safety data without reactogenicity (Bharat Biotech’s Covaxin shows 78% efficacy in Phase 3 analysis). All the side effects combined were observed to be about 15%. As per the vaccine manufacturers, the third phase trials would possibly cover around 28 500 of subjects from 18 years of age and above [181]. On 3 January, covaxin got its emergency authorization in India and it was found that Covaxin is up to 70% efficacious against mild, moderate and severe COVID-19, and 100% efficacious for severe COVID-19 especially, due to the upsurge in COVID-19 cases in April in India, and scientists found that covaxin is discreetly less efficient against the new variant named B.1.617 or G/452R.V3. Although it does not mean that the vaccine is not capable of giving fortification against the virus.

#### Sinopharm-BIBP COVID-19 vaccine (BBIBP-CorV)

The Beijing Institute of Biological Products collaborated with a state-owned Chinese company called Sinopharma and created an inactivated COVID-19 vaccine that was put onto controlled clinical trials. On 30 December 2020, Sinopharm reported that the vaccine efficacy rate is 79.34%, making the Chinese government give it a green flag. The company later made a contradictory remark by stating that the efficacy rate is 72.51% although it has yet to publish the data of their Phase 3 trial. Two doses of the vaccine need to be given 3 weeks apart from each other. The whole COVID-19 virus was cultivated *in vitro* in a cell line, and the infected cells were further inactivated twice by the help of β-propiolactone under and further adsorbed to 0.5 mg alum. The Phase 1 and Phase 2 trials suggested that with a longer interval time period up to 21 days between the first dose and second booster dose, it produces higher antibody titers in comparison to shorter interval time period which were 14-day schedules [181].

Some excellent work has already been done and yet there is so much to explore in this journey of finding the safest, efficacious as well as the most economical option for COVID-19 vaccine. More information in regard to immunization route, finding more target antigen(s) would certainly help in overcoming this pandemic with a greater speed, also other aspects like its manufacturing, stability, side effects and global access should be kept in mind. Various COVID-19 vaccine candidates are going through clinical trials and some of them are yet to publish the Phase 3 trial results; the third phase trials are a very crucial aspect in making the COVID-19 vaccine since it establishes the safety and its effectiveness at a large scale with different types of population (Table 1). Several long-term studies are undergoing to assess the impact of COVID-19 vaccination on diverse populations globally. Further long-term assessment is required to investigate long-term immunity from viral infections. Approved vaccines and their booster doses can lead to protective immune responses. However, further evaluation is required to target candidates with augmented protective coverage against emerging variants.

**Table 1.** List of COVID-19 vaccine candidates

Vaccine company/Name	Vaccine platform
Pfizer-BioNTech	mRNA vaccine
Moderna	mRNA vaccine
Sputnik-V	Vector vaccine
Oxford–AstraZeneca (covishield)	Vector vaccine
CanSino Biologics’ Convidecia	Vector vaccine
Johnson & Johnson’s vaccine	Vector vaccine
Bektop—EpiVacCorona	Protein-based vaccine
Sinopharm—BBIBP-CorV	Inactivated vaccine
Bharat Biotech-Covaxin	Inactivated vaccine
Sinovac Biotech-CoronaVac	Inactivated vaccine
Curevac—CVnCoV	mRNA vaccine
Zydus Cadila: ZyCoV-D	DNA vaccine
AnGes—AG0302-COVID-19	DNA vaccine
CanSinoBIO- Convidecia	Vector vaccine
Novavax	Protein-based vaccine
Soberana 2	Protein-based vaccine
Medicago-CoVLP	Protein-based vaccine
Clover Biopharmaceuticals	Protein-based vaccine
Institute of Medical Biology	Inactivated vaccine
RIBSP vaccine	Inactivated vaccine
INOVIO-INO-4800	DNA-based vaccine
Arcturus Therapeutics—Duke-NUS Medical School	mRNA vaccine
Israel Institute for Biological Research—Brilife	Protein-based vaccine
West China School of Medicine	Protein-based vaccine
Medigen—Dynavax	Protein-based vaccine
Centre for Genetic Engineering and Biotechnology, Cuba	Protein-based vaccine
Covaxin	Protein-based vaccine
Sanofi	mRNA vaccine
Erciyes University	Inactivated vaccine
Biokangtai	Inactivated vaccine
Abogen—Walvax	mRNA vaccine
Chula Vaccine Research Center	mRNA vaccine
Entos Pharmaceuticals	Protein-based vaccine
Symvivo	DNA vaccine
Oncosec Immunotherapies	DNA vaccine
Providence Therapeutics	mRNA vaccine
Takis Biotech—Rottapharm Biotech	DNA vaccine
Vaxart	Vector vaccine
DZIF	Inactivated vaccine
ImmunityBio	Vector vaccine
City of Hope	Inactivated vaccine
Cellid	Vector vaccine
Altimmune	Vector vaccine
Bharat Biotech- BBV154	Vector vaccine
Ichan School of Medicine	Inactivated vaccine
Gritstone Oncology	Protein-based vaccine
KBP Kentucky Bioprocessing	Protein-based vaccine
University of Tubingen	Protein-based vaccine
SK Bioscience	Protein-based vaccine
Nanogen Biopharmaceuticals	Protein-based vaccine
Vido-Covac	Protein-based vaccine
Riza	Protein-based vaccine

## Conclusions and future perspective

The emergence of SARS-CoV-2 and resultant COVID-19 pandemic has brought immense difficulties worldwide. As a result of extraordinary scientific efforts to counteract the most fatal pandemic of the 21 century, we are rationally progressing from these adverse circumstances. Identification, isolation and sequencing of SARS-CoV-2 shortly after the outbreak enabled the timely development of diagnostics for COVID-19 detection. Timely diagnosis and immediate isolation of infected individuals effectively restricted SARS-CoV-2 transmission and saved numerous lives. Additionally, early prognosis and detection of COVID-19-associated complications assisted in employing appropriate therapeutic approaches to improve clinical outcomes and prevent mortality. The momentum at which research was conducted and published assisted in understanding the biology of SARS-CoV-2. Information on structural organization and immune responses was harnessed to develop several therapeutics and vaccines against SARS-CoV-2. The utilization of knowledge acquired from prior coronavirus outbreaks and repurposing of various pre-existing drugs has significantly relieved the situation in many countries. While the existence of COVID-19 is continuously transforming with the emergence of new VOC, in this review, we have reassessed current knowledge regarding SARS-CoV-2 that is paramount to establish suitable therapeutics of COVID-19. Since, ending the pandemic will necessitate the implementation of suitable therapies, vaccination strategies, detailed studies on immune response and application of diagnostic testing to halt transmission of SARS-CoV-2, we must aim to deal with the shortcomings of existing approaches and improvise for robust clinical trajectories.

## Ethics approval

No ethical approval was required for this study.

## Data Availability

We hereby declare that this review will be openly available for all.
